# Identifying Nutrient Patterns in South African Foods to Support National Nutrition Guidelines and Policies

**DOI:** 10.3390/nu13093194

**Published:** 2021-09-14

**Authors:** Yusentha Balakrishna, Samuel Manda, Henry Mwambi, Averalda van Graan

**Affiliations:** 1 Biostatistics Research Unit, South African Medical Research Council, Durban 4001, South Africa; 2 School of Mathematics, Statistics and Computer Science, University of KwaZulu-Natal, Pietermaritzburg 3201, South Africa; samuel.manda@mrc.ac.za (S.M.); mwambih@ukzn.ac.za (H.M.); 3 Biostatistics Research Unit, South African Medical Research Council, Pretoria 0001, South Africa; 4 Department of Statistics, University of Pretoria, Pretoria 0001, South Africa; 5 Biostatistics Research Unit, SAFOODS Division, South African Medical Research Council, Cape Town 8001, South Africa; averalda.vangraan2@mrc.ac.za; 6 Division of Human Nutrition, Department of Global Health, Stellenbosch University, Cape Town 8001, South Africa

**Keywords:** food composition database, nutrient pattern, nutrient composition, principal component analysis, food-based dietary guideline, salt intake, South Africa

## Abstract

Food composition databases (FCDBs) provide the nutritional content of foods and are essential for developing nutrition guidance and effective intervention programs to improve nutrition of a population. In public and nutritional health research studies, FCDBs are used in the estimation of nutrient intake profiles at the population levels. However, such studies investigating nutrient co-occurrence and profile patterns within the African context are very rare. This study aimed to identify nutrient co-occurrence patterns within the South African FCDB (SAFCDB). A principal component analysis (PCA) was applied to 28 nutrients and 971 foods in the South African FCDB to determine compositionally similar food items. A second principal component analysis was applied to the food items for validation. Eight nutrient patterns (NPs) explaining 73.4% of the nutrient variation among foods were identified: (1) high magnesium and manganese; (2) high copper and vitamin B_12_; (3) high animal protein, niacin, and vitamin B_6_; (4) high fatty acids and vitamin E; (5) high calcium, phosphorous and sodium; (6) low moisture and high available carbohydrate; (7) high cholesterol and vitamin D; and (8) low zinc and high vitamin C. Similar food patterns (FPs) were identified from a PCA on food items, yielding subgroups such as dark-green, leafy vegetables and, orange-coloured fruit and vegetables. One food pattern was associated with high sodium levels and contained bread, processed meat and seafood, canned vegetables, and sauces. The data-driven nutrient and food patterns found in this study were consistent with and support the South African food-based dietary guidelines and the national salt regulations.

## 1. Introduction

Public health nutrition focuses on promotion and improvement of optimal health of a population through nutrition-related health dietary guidelines and policies. In the sub-Saharan African region, public health challenges such as the increasing burden of malnutrition, diabetes mellitus and cardiovascular diseases, can potentially be addressed with adequate nutrition interventions [1,2]. However, to implement effective nutritional interventions in the region, the nutritional situation of the targeted population needs to be known. This requires reliable food consumption data.

Food composition databases (FCDBs) are essential to public health nutrition and associations between diet and health have been shown at the levels of dietary patterns, food groups, foods, and nutrients [3]. They are used together with dietary intake studies to develop food frequency questionnaires and assess relationships between diet and disease. FCDBs also provide insight into food groups and foods containing low or high nutrient levels. Once these relationships have been determined, food-based dietary guidelines (FBDGs) and nutrition policies can be implemented. FBDGs translate recommended dietary allowances to food-related guidelines for improved public health nutrition and guidance [4]. South Africa first developed the FBDGs in 2003 and revised the guidelines in 2012. The South African FBDGs have since been adopted by the National Department of Health as the ‘official’ dietary recommendations for the country in people aged 5 years or older [4]. The eleven guidelines aim to promote a change in the dietary habits of South Africans to address nutrition-related public health diseases such as malnutrition and obesity. The guidelines encourage dietary diversity and highlight foods that should be limited such as fats, sugar, and salt. Other public health nutrition measures to improve health such as food fortification [5], salt regulations [6] and taxes on sugar-sweetened beverages [7], have also been implemented in South Africa.

Fruits, vegetables, legumes, dairy, and meat are just a few of the common food groups found in FCDBs and accepted by nutritionists. Food items within these food groups generally provide similar amounts of macronutrients. However, while nutritional composition may be similar within these groupings, subgroups may be identifiable and compositional similarity may also be found across these groupings. The growing number of food items in FCDBs presents consumers with dietary choices that need to be based on nutrition, availability, cost, and preference. Classifying food items into nutritionally homogenous groups allows consumers to select alternative food items whilst maintaining a similar nutritional intake. Identifying compositionally similar food items guides dietary recommendations, assists in consumer education, and informs product reformulation. With the ever-expanding food market and inclusion of country-specific foods, it can also aid the categorization of a new food item by grouping it with similar foods that are already known [3]. The identification of unhealthy food items that may not be immediately apparent, also becomes possible.

Several studies have investigated the clustering of food items [8,9,10,11,12] and nutrient co-occurrence patterns [13,14] using statistical methods, but only one was found to use data from Africa [15]. More specifically, the study of nutrient patterns in South Africa has been limited to consumption data [16,17,18,19]. Thus, there is a need to develop capacity in methods applicable to the African scenario to help inform consumers and public health policy makers in food nutrient patterns and composition.

Using statistical methods, this study aims to identify compositionally similar food items and nutrient co-occurrence patterns within the South African Food Composition Database (SAFCDB) [20]. The results of this study will provide data-driven evidence that may support the current dietary guidelines and nutritional policies or offer an alternative view.

## 2. Materials and Methods

### 2.1. Data

The 2017 SAFCDB [20] (available at http://safoods.mrc.ac.za/products.html, accessed on 9 September 2021) contained nutrient information on 1667 food items and 169 food components. This consisted of both uncooked and cooked food items, as well as composite dishes. Fortified food items were described as such. Table 1 provides a detailed description of the food items by food group. For ease of reference, we will use the term ‘nutrients’ to encompass the nutrients, minerals and vitamins used in the analysis. All nutrient values were expressed per 100 g. The most common nutrients with a minimal quantity of missing values were selected for analysis (*n* = 28; Table 2). In our selection of the nutrients, we also ensured that nutrients were non-collinear. For example, because total carbohydrate is the sum of available carbohydrate and dietary fibre, we opted to include available carbohydrate and dietary fibre instead of total carbohydrate. Nine macronutrients, nine minerals, and ten vitamins were analysed. Due to the standard principal component analysis (PCA) technique requiring complete data for all variables, all food items that had complete nutrient information for the selected 28 nutrients were included in the principal component analysis (*n* = 971).

### 2.2. Methods

**Figure 1:**
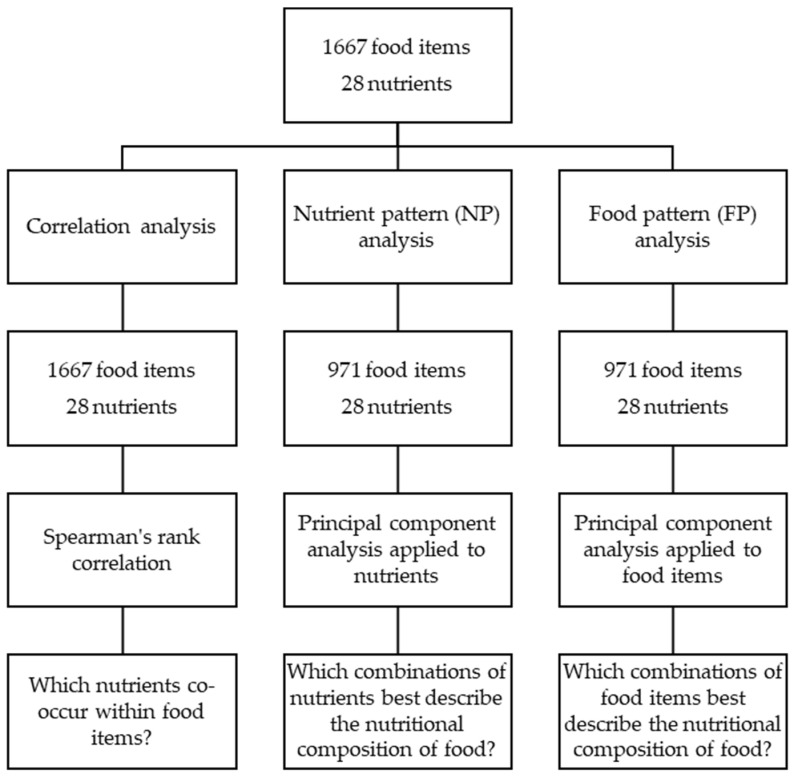
Flow chart of methodology and rationale.

Statistical methods that consider the correlated nature and presence of multiple nutrients within a food item are needed to evaluate the nutrient patterns amongst food items. Principal component analysis is one of the oldest and simplest dimension-reduction techniques available [21] and is applicable to correlated variables. When applied to food composition data, PCA allows the analysis of multiple nutrients simultaneously. PCA aims to describe the maximum amount of variation in the dataset using the least number of principal components (PCs). The PCs are uncorrelated linear combinations of the original variables that capture most of the variation within the first few components. PCA aids data reduction by explaining the covariation amongst the variables using a few linear combinations. PCA also aids data interpretation by finding features that explain the covariation. The contribution of each variable to a component is called the loading and high loadings indicate important variables. Rotation methods can be applied to enhance interpretability by producing loadings that are as close to zero or one as possible. For each PC, observations have a score that combines each of the variables. The score indicates how much each observation is related to a PC [22]. Factor analysis is also a common multivariate dimension reduction technique but has slight differences to PCA. While PCA describes the relationships among the observed variables in a simpler way, factor analysis finds latent factors that influence the observed variables. Hence, the application of factor analysis is more suited to the analysis of consumption data as it will be able to generate latent factors, that is, dietary patterns, which predict food choices [23]. Figure 1 presents the methodology and rationale.

#### 2.2.1. Correlation Analysis

For each nutrient, some foods contained exceptionally high values. For example, oysters were especially high in zinc and amaranth leaves were especially high in magnesium. Due to these outliers, we calculated pairwise-complete Spearman correlations for the complete dataset (*n* = 1667), to determine nutrient co-occurrence patterns.

#### 2.2.2. Nutrient Pattern Analysis

For the sub-sample (*n* = 971), we explored the data using PCA with orthogonal varimax rotation and Kaiser normalization to enhance interpretability. PCA was applied to the correlation matrix due to the scale differences between nutrients. Components were retained considering the scree plot, eigenvalues greater than 1 (the average of the eigenvalues when using the correlation matrix) and interpretability. High-loading nutrients were defined as having an absolute loading of at least 0.4 and were used to interpret the component. To enhance and support the interpretation, nutrients with absolute loadings between 0.3 and 0.4 were also considered. Food items were allocated to groups corresponding to their highest PC score. The chi-square test was used to test for an association between the FCDB and PCA groupings. The Kruskal–Wallis test was used to test for differences in nutrient values between the PC groupings. The PCs identified by the nutrient analysis were termed ‘nutrient patterns (NPs)’.

#### 2.2.3. Food Pattern Analysis

We also applied PCA to the food items to confirm the components found during the nutrient pattern analysis. Components with eigenvalues greater than 1 and that accounted for at least 1% of the variation were retained. The highest absolute loading within each component ranged between 0.07 and 0.16. Hence, absolute loadings greater than 0.05 were used to interpret the component. The PCs identified by the food item analysis were termed ‘food patterns (FPs)’.

Trace values (values below the limit of detection) accounted for 1.2% of the data and were imputed with half of the limit of detection for each nutrient [24]. Results were considered significant for *p* < 0.05 and Bonferroni-adjusted significance levels were used to account for multiple testing. All analyses were done in Stata version 16 (StataCorp, College Station, TX, USA) and R (available at https://www.R-project.org/, accessed on 9 September 2021).

## 3. Results

### 3.1. Correlation Analysis

**Figure 2:**
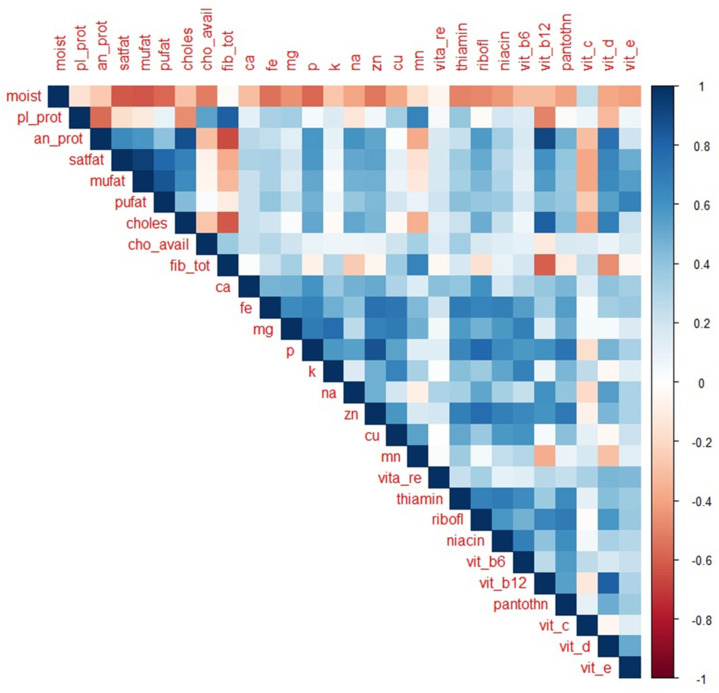
Spearman’s rank correlations between the nutrients.

The correlations between the nutrients are presented in Figure 2. Overall, correlations were mostly positive indicating frequent nutrient co-occurrences. Negative correlations occur when the increase in one nutrient results in the decrease of another nutrient. Most negative correlations were found between moisture and all other nutrients, except vitamin C (r = 0.25, *p* < 0.001). Animal protein, fatty acids, and cholesterol positively correlated with phosphorous, sodium, zinc, riboflavin, vitamin B_12_, pantothenic acid and vitamin D (*p* < 0.001). In contrast, animal protein, saturated fatty acids, mono-unsaturated fatty acids, and cholesterol, negatively correlated with total fibre and vitamin C (*p* ≤ 0.001). Vitamin E had the highest positive correlations with fatty acids (r = 0.46, r = 0.52, r = 0.67, *p* < 0.001) and vitamin D (r = 0.64, *p* < 0.001). Plant protein had the highest positive correlations with total fibre (r = 0.82, *p* < 0.001) and manganese (r = 0.67, *p* < 0.001). Plant protein and total fibre both negatively correlated with animal protein, cholesterol, and vitamin B_12_ (p < 0.001). Vitamin B_12_ and vitamin D exhibited similar patterns, both negatively correlating with plant protein, total fibre, and manganese (*p* < 0.001). Strong, positive correlations among the minerals and vitamins were also found. Iron, magnesium, and copper were connected by positive correlations (*p* < 0.001) as well as thiamin, riboflavin, niacin, and vitamin B_6_ (*p* < 0.001). Positive correlations were also evident between animal-derived micronutrients such as phosphorus, zinc, and pantothenic acid (*p* < 0.001).

### 3.2. Nutrient Pattern Analysis

Eight nutrient patterns had an eigenvalue greater than 1 (Figure S1) that explained 73.4% of the total nutritional variation in the data. The rotated loadings are presented in Table A1 in Appendix A. A characterisation of the patterns, using the nutrients that loaded highly (absolute loadings >0.4) on each, is shown in Table 3, along with the supporting nutrients that had absolute loadings between 0.3 and 0.4. At least two nutrients per pattern had high loadings. PC scores were calculated for each food item and the highest score determined pattern membership. NP 1 was characteristic of food items high in plant protein, total fibre, magnesium, potassium, and manganese. Iron also featured on NP 1 but had a loading of 0.27—less than our threshold of 0.3. Wheat products, dark leafy greens, legumes, nuts, and seeds scored highest on this pattern. NP 2 was found to be high in vitamin A, copper, riboflavin, and vitamin B_12_ and linked with foods such as kidney, liver, mussels, and oysters. Meat, meat products, crab, and oily fish scored high on NP 3 as they shared high levels of animal protein, niacin, and vitamin B_6_. Fortified bread was also included due to its increased vitamin B_6_ content. Saturated fatty acids, mono-unsaturated fatty acids, polyunsaturated fatty acids, and vitamin E characterised NP 4 and were found to be highest in fats and oils, avocados, some nuts (almonds, pecans, walnuts, macadamias, and coconuts), and sauces. Foods made or fried with oil or margarine also scored highly on this pattern, as well as chicken skin and processed meats. NP 5 identified foods high in calcium, phosphorous and sodium such as milk, milk products, canned vegetables, biltong, and shrimp. Foods made with milk and cheese were also found to be associated with NP 5. While most nutrients had positive loadings, moisture and zinc had a negative loading on NPs 6 and 8, respectively. NP 6 had positive loadings of available carbohydrate and thiamin, correlating with baked items, dried fruit, jams, as well as sugar and sweets, while NP 8 had positive loadings of vitamin A and vitamin C. Fruits and vegetables related mostly to NP 8, as well as soft maize meal. High cholesterol and vitamin D content characterised NP 7. Foods associated with this pattern were eggs, composite dishes using eggs, fish, offal, and tripe. Fortified milk powder and breastmilk substitutes also scored high on this nutrient pattern. Pantothenic acid featured on NP 2 and NP 7 but, like iron, had loadings below the absolute value threshold of 0.3.

Table 4 compares the food categories in the SAFCDB to the groupings found by the PCA. PCA groupings consisted of food items across the SAFCDB groupings, and the grouping structures were significantly associated (*p* < 0.001). Vegetables and legumes contributed 42% and 23% of NP 1, respectively. Meat and seafood together accounted for 91.8% of NP 3. All food items in the category ‘Eggs’ were grouped under NP 7, together with composite dishes from ‘Cereals and cereal products’ that were made with eggs. Most of the food items within ‘Legume and legume products’, ‘Milk and milk products’, ‘Fats and oils’ and ‘Sugar, syrups and sweets’ remained together under the PCA groupings.

**Figure 3:**
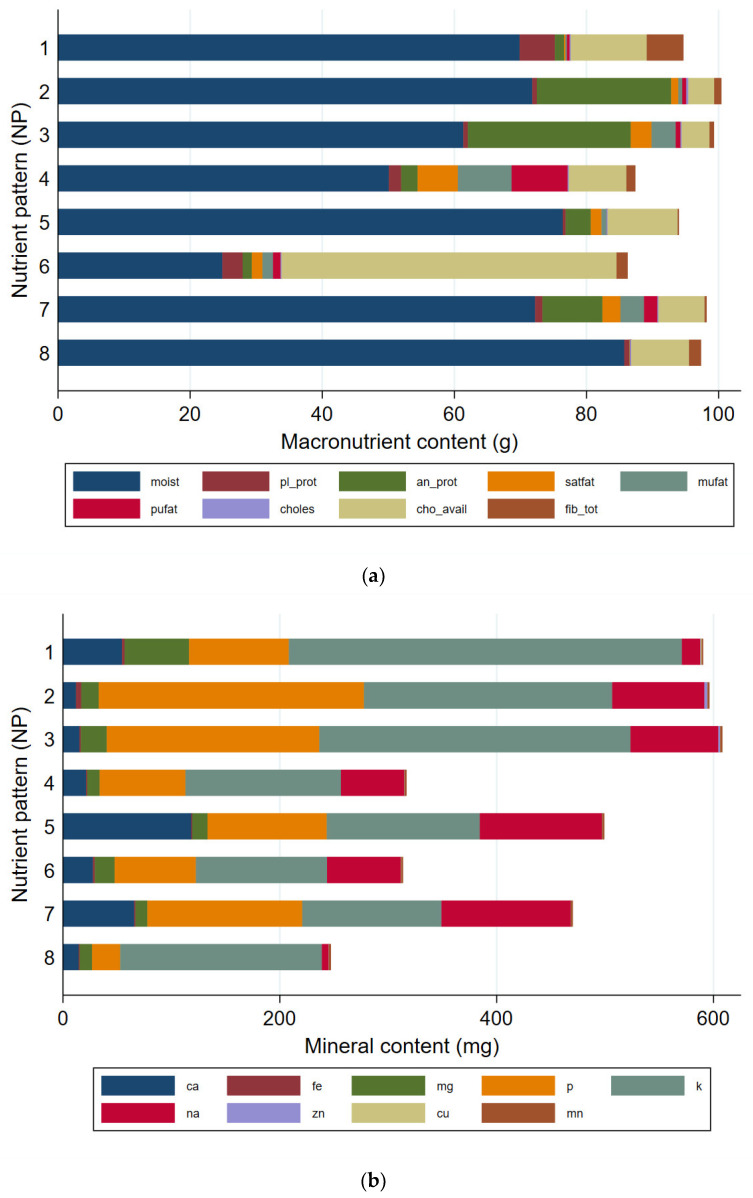

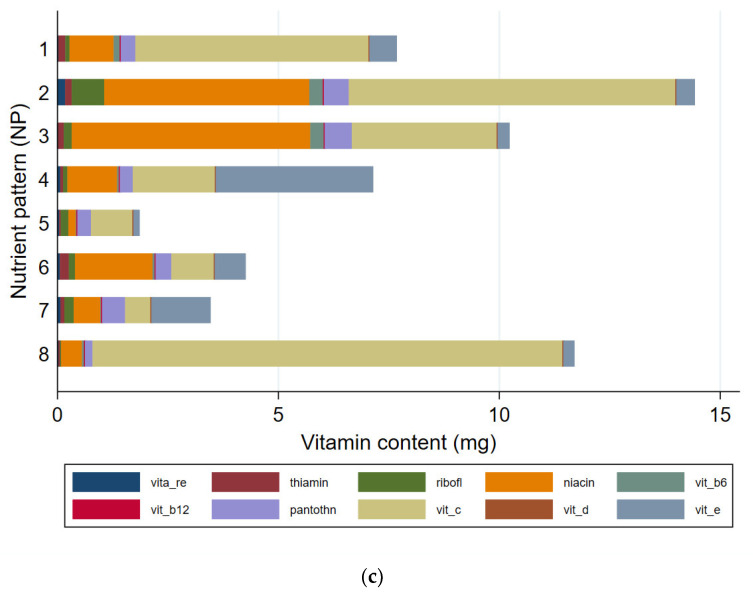
Median nutrient values per nutrient pattern (NP) for (**a**) macronutrients; (**b**) minerals and (**c**) vitamins.

Table A2 in Appendix A reports the median (IQR) nutrient values for each principal component grouping. Median nutrient values for each NP agreed with the characterisation of the patterns and are graphically represented in Figure 3. Table A2 in Appendix A and Figure 3 represent the expected nutritional composition of an average food item from each pattern. A randomly selected food item from NP 3 will, on average, contain the highest amount of animal protein and niacin than a food item from any of the other NPs. Sodium content can be expected to be lowest in foods from NP 8, and highest in foods from NP 5 and NP 7. NP 2 and NP 8 had the greatest cumulative quantity of vitamins, with vitamin C contributing the largest proportion of the composition. Niacin was also a large contributor of vitamin content in NP 2. The first three patterns had the highest quantity of minerals made up largely from phosphorous and potassium.

### 3.3. Food Pattern Analysis

We also applied PCA to the 971 food items to confirm the nutrient patterns we found. Seven food patterns had both an eigenvalue greater than 1 and accounted for at least 1% of the variation in the dataset (Figure S2). The seven FPs explained 97.4% of the total variation in the data (Table 5). The maximum absolute loading for components ranged from 0.07 to 0.16, hence, we interpreted the components using absolute loadings that were greater than 0.05. The patterns found when analysing the food items reflected the patterns found when analysing the nutrients thus, confirming the presence of nutrient patterns and validating our results. Both analyses identified a pattern grouping together wheat products, leafy vegetables, and legumes (NP 1 and FP 2) as well as patterns for milk and milk products (NP 5 and FP 3), and eggs and food items using eggs (NP 7 and FP 4). However, applying PCA to the food items enabled better discrimination of fruit and vegetables by vitamin C (FP 1) and beta-carotene (FP 6) content. Orange-coloured fruit and vegetables were identified with FP 6 in the food item analysis. In addition, greater discrimination was apparent among dark leafy greens, which were split between FP 2 and FP 6 compared to being grouped together under the nutrient analysis. Composite dishes using distinctive ingredients were also able to be identified and grouped with the raw versions of the ingredient. For example, carrot cake grouped with carrots and pastries made using eggs grouped with eggs. Rusks made with wholewheat flour (FP 2) and rusks made with white flour (FP 5) were also able to be identified and separated. Processed meat such as luncheon meat and sausages were separated from meat and meat products, and instead grouped with processed cheese (FP 5) and processed fish. Sodium scored high on this food pattern. The last pattern in the food item analysis (FP 7) separated soft maize meal from the stiff and crumbly versions based on its higher moisture content, similar to the results of the nutrient analysis. Soft maize meal was grouped together with other moisture rich food items such as beverages, cabbage and brinjal.

The patterns found supported the South African FBDGs [4], as shown in Table 6. Guideline 1 aims to facilitate balanced nutrient intake by encouraging the consumption of a variety of foods. As the nutrient patterns obtained differ in nutritional composition, consuming foods from different patterns supports this guideline. Starchy foods, as described in Guideline 2, such as bread, rice, cereals, and pasta were associated with NP 6. NP 6 also contained products high in sugar content such as cakes, cookies, and sweets and reflects Guideline 10. Similarly, other nutrient patterns were able to be matched to the South African FBDGs.

Guideline 11 was best captured by FP 5 and reflected categories targeted by the national sodium regulation [6], as highlighted in Table 7. Foods affected by the regulation were all found within FP 5.

## 4. Discussion

Public health practitioners and policy makers rely on FCDBs to assess nutrient availability and provide information to link dietary data with nutrient intake for nutritional epidemiology. They also utilize FCDBs for developing nutrition interventions and for informing consumer education. Policies impact food product composition to address dietary shortfalls, but the full potential of food composition is often not recognized [25]. In South Africa, studies have been limited to determining consumption habits among populations [16,17,18,19] but our study aims to examine the nutrient patterns present within the food items consumed by the population. More specifically, we aimed to examine the nutrient patterns present among food items listed in the SAFCDB [20] using correlation and PCA. FCDBs are often country-specific due to the influence of environmental, genetic, and processing factors on the nutrient content of food. National FCDBs also include country-specific foods and recipes, reflecting the unique consumption patterns of the country [26]. Therefore, analysing foods contained in the SAFCDB would provide information on the nutrient levels of foods consumed by the South African population.

Significant correlations between the nutrients were identified. Nutrients obtained primarily from plant-based foods, such as total fibre and available carbohydrates, exhibited a strong positive correlation with plant protein. Nutrients obtained primarily from animal products, such as cholesterol and vitamin B_12_, were strongly associated with animal protein. These plant-derived nutrients negatively correlated with animal-derived nutrients, confirming what is known about nutrient co-occurrence. Our results are also consistent with the correlations found elsewhere among raw foods [13] and raw plant foods [9], suggesting that similar nutrient patterns are evident among cooked and composite dishes as well, which were included in our analysis. The underlying correlation structure contributes to features that distinguish between nutrient-based food groupings. This must be accounted for in any statistical analyses undertaken using multivariate methods. In addition, the high correlation implies better prediction models which are useful in estimating values of missing nutrients, a problem common to FCDBs. While the 2017 SAFCDB contained nutrition data for 1667 food items, only 971 food items could be analysed due to missing data. In addition, missingness also excluded biotin and folate from the analysis, which are both vital B-vitamins that are sourced from food [27]. Methods to impute missing values in food composition data have been investigated [28,29,30] and further research in this area could facilitate the completeness of FCDBs.

Our study affirmed that some food items are more compositionally alike than others, by identifying eight nutrient patterns that were consistent with existing knowledge. All analysed nutrients, except iron and pantothenic acid, featured on a pattern. Although iron and pantothenic acid did not meet our threshold for a high loading, both stood out on nutrient patterns that contained their expected sources. Vitamin A featured on two nutrient patterns, due to its availability in foods of both plant and animal origin. A study [14] conducted in Finnish foods, identified four nutrient content patterns using factor analysis and was able to group wheat products with legumes, and mushrooms with offal foods—a common finding in our study as well. Although the study was able to include 106 nutrients, the patterns were comparable to the patterns found in our study, suggesting that only a few key nutrients are needed to successfully determine nutrient patterns.

We also validated our results by applying the dimension reduction technique to the food items themselves. Results of both analyses were similar, and a large amount of the nutritional variation was able to be explained by a few patterns. The patterns included food items from across different food groups, suggesting compositional similarity despite conceptual dissimilarity. Hence, applying clustering techniques within each conceptual FCDB group may reveal more intricate groupings. However, this approach may suffer from high dimensionality with small sample size issues. Two studies applied clustering techniques within FCDB food groups. The first study [15] found six subgroups within the ‘Cereals’ category of the West Africa Food Composition Table. These subgroups separated grains by type and preparation methods. For example, pearl millet separated from other grains, and maize was separated across three clusters depending on whether it was raw, boiled, or prepared as a porridge. Likewise, our analysis differentiated between white and brown rice, and soft maize meal and stiff or crumbly maize meal. The second study [9] applied clustering techniques within five food categories (fruits, vegetables, nuts and seeds, legumes, and cereal grains) of the U.S. Department of Agriculture (USDA) National Nutrient Database for Standard Reference (SR) Legacy (2018). The study found that similar foods were not necessarily from the same category. For example, wheat germ was found to cluster with legumes, a finding repeated in our analysis as well. Another similar finding was almonds and coconuts, macadamias, pecans, and walnuts separating from other nuts in the database. Chestnuts were also isolated from other nuts. Our results suggest that statistical methods can be used to create a natural food exchange list to accommodate different dietary preferences.

Dark leafy greens such as spinach and other leaves (amaranth, blackjack, cowpea, etc.) were differentiated from other vegetables in the database. The application of PCA to food items had greater discernability than PCA applied to nutrients. Under the food pattern analysis, dark leafy greens were further divided into spinach and amaranth leaves and other leafy greens. Similarly, orange-coloured fruit and vegetables grouped together, which was not seen under the nutrient pattern analysis. This type of clustering was also identified in Pennington et al. [10]. The daily consumption of dark-green leafy vegetables and orange-coloured fruit and vegetables is recommended as per the South African FBDGs [4] and the Dietary Guidelines for Americans [31] and is important for a healthy diet as they are rich sources of vitamins and minerals [4]. Classifications that are based on nutritional similarity are useful to nutritionists, researchers, and consumers for the development of dietary guidance materials, development of food frequency questionnaires and reporting of consumption studies, and adherence to dietary guidelines [32].

The PCA method was also able to separate canned vegetables and vegetables fried in oil from the other vegetables. This is helpful in determining food preparation characteristics from nutrient information. Both analyses were able to identify foods made with egg, such as choux pastry and custard, and group these items together with eggs. However, the nutrient analysis additionally included milk and savoury tarts, which are traditionally made with egg. Similarly, both analyses were able to identify foods made with milk and cheese, such as malted milk beverages, puddings, yoghurt, and cheese sauces. Employing a principal component analysis may additionally be helpful in identifying ingredients for composite dishes in a FCDB.

Our results provide data-driven evidence to support the existing knowledge of food and nutrient patterns, as well as South African food-based dietary guidelines and nutrition policies. Each of the nutrient patterns identified corresponded to a guideline and supports the consumption of a variety of foods and moderation of other foods. High sodium levels in food items have led to the current promulgated salt regulation and reduction of salt content of food items in the country [6]. Food items belonging in the high sodium food pattern closely mirrored the categories identified in the regulation. Under the food item analysis, canned vegetables grouped together with other processed food items on a high sodium pattern. Canned vegetables, processed meat, processed cheese, bread, and sauces are suggested to have similar levels of sodium, and this is consistent with research showing these categories to have the highest median sodium levels, based on packaged foods in South Africa [33]. This analysis supports the regulation and can be used in a similar fashion to identify foods with a high sugar content. FBDGs are developed in response to a public health problem [34] and requires identifying rich sources of nutrients that are of public health importance [35]. The patterns identified in our results each describe foods that are rich sources of specific nutrients. Foods providing these nutrients are recommended to be either limited or increased, as appropriate, and implementation of the FBDGs should then be accompanied by monitoring and evaluation of the effects. Food systems [36] are dynamic and are influenced by key drivers such as regulatory frameworks, consumer influence, technological innovations, concerns for food safety, and growing attention paid to diet and health [37,38]. Thus, continuous updates of a FCDB are essential to reflect the changes not only in the types of food provided but also the composition thereof [39]. The evaluation of the effects can be based on changes in food composition [34] and some studies have applied statistical methods to different versions of FCDBs to determine changes over time in composition of fruits and vegetables [40,41,42]. Therefore, repeating our analysis on past versions and future updates of food composition data could assess whether the implementation of FBDGs and regulations have impacted the reformulation of products. The SAFCDB is updated every three years as updates are resource-intensive and can be challenging to regularly implement, as updates are applied to all database-related tools and products such as publications, software programs, and applications.

The research of innovative statistical methods tailored towards food composition data has the potential to provide improved evidence for dietary guidelines and policy. In addition, it can also support the dietary patterns found in consumption studies. Makura-Kankwende et al. [17] showed that the animal driven dietary pattern, characterised by animal protein and saturated fat, was associated with an increased body mass index amongst black South African women. From our results, foods high in animal protein and saturated fat correspond to meat and meat products, processed meat, and fried foods. These foods are generally present in the Western diet, and the animal driven pattern found may be suggestive of a shift towards this diet [17]. Another study, Visser et al. [19] found that a dietary pattern featuring vitamin A and vitamin B_12_ was associated with lower odds of anaemia in 5–12-year-old South African children. This dietary pattern is reflected in our results which identified foods containing this combination of nutrients, mainly, organ meat such as kidney and liver [19].

Some limitations need to be considered. Missing nutrient values excluded essential nutrients such as folate and biotin from the analysis and contained our sample to 58% of foods available in the SAFCDB. With respect to the PCA method, subjective decisions on the data matrix, rotation method, number of retained components and loading threshold need to be made [16,18]. However, our results are consistent with existing knowledge and has strengths in presenting nutrient and food patterns among South African foods that support food-based dietary guidelines, nutrition policies and consumption studies. We are currently working on developing K-Means and Gaussian Mixtures (GMs) clustering models to identify food items that are more like each other. We are aware that several food items contain missing nutrient values in the database, so we will incorporate multiple imputation techniques to account for missing data. We believe the development and application of these models to food composition databases will contribute to an understanding of nutritional uptake in the population and monitoring adherence to national nutritional prevailing regulation and guidelines.

## 5. Conclusions

To our knowledge, this is the first study to investigate nutrient patterns in food items using South African food composition data. This analysis provides an overview of the inherent groups available within South African foods. The nutrient co-occurrence patterns identified using data-driven methods are consistent with current knowledge and comparable to similar studies from other countries. The results support current dietary guidelines and nutritional policies.

## Figures and Tables

**Table 1:** Number of foods per food group.

Food Group	*n*	%
Cereals and Cereal Products	273	16.38
Vegetables	312	18.72
Fruit	143	8.58
Legumes and Legume Products	37	2.22
Nuts and Seeds	27	1.62
Milk and Milk Products	76	4.56
Eggs	30	1.80
Meat and Meat Products	172	10.32
Fish and Seafood	61	3.66
Fats and Oils	50	3.00
Sugar, Syrups and Sweets	48	2.88
Soups, Sauces, Seasonings and Flavourings	76	4.56
Beverages	52	3.12
Infant and Paediatric Feeds and Foods	250	15.00
Therapeutic/Special/Diet Products	32	1.92
Miscellaneous	28	1.68
Total	1667	100.00

**Table 2:** Nutrients included in the analysis with their unit of measurement and corresponding abbreviations used in figures.

Macronutrients	Minerals	Vitamins
Moisture (g), moist	Calcium (mg), ca	Vitamin A (RE) (μg), vita_re
Plant protein (g), pl_prot	Iron (mg), fe	Thiamin (mg), thiamin
Animal protein (g), an_prot	Magnesium (mg), mg	Riboflavin (mg), ribofl
Saturated fatty acids (g), satfat	Phosphorous (mg), p	Niacin (mg), niacin
Mono-unsaturated fatty acids (g), mufat	Potassium (mg), k	Vitamin B_6_ (mg), vit_b6
Polyunsaturated fatty acids (g), pufat	Sodium (mg), na	Vitamin B_12_ (μg), vit_b12
Cholesterol (mg), choles	Zinc (mg), zn	Pantothenate (mg), pantothn
Carbohydrate, available (g), cho_avail	Copper (mg), cu	Vitamin C (mg), vit_c
Total dietary fibre (g), fib_tot	Manganese (μg), mn	Vitamin D (μg), vit_d
		Vitamin E (mg), vit_e

Abbreviations: g = grams; mg = milligrams; μg = micrograms; RE = retinol equivalents.

**Table 3:** Characterisation of nutrient patterns (NP).

NP	Nutrients with Absolute Loadings >0.3 and >0.4	Examples of Food Items That Scored Highly on Pattern
1	high in plant protein, total fibre, *magnesium*, potassium, and *manganese*	wheat products, oats, brown rice, dark leafy greens, peas, dehydrated green beans, dehydrated cabbage, dehydrated cauliflower, legumes and legume products, nuts, seeds
2	high in vitamin A, *copper*, riboflavin, and *vitamin B_12_*	kidney, liver, giblets, mussels, oyster, mushroom
3	high in *animal protein*, *niacin*, and *vitamin B_6_*	meat and meat products, crab, oily fish, fortified bread/rolls
4	high in *fatty acids* and *vitamin E*	foods made or fried with oil or margarine, chicken skin, processed meats, fats and oils, avocado, nuts, sauces
5	high in *calcium*, *phosphorous*, and *sodium*	milk and milk products (including foods made with milk and cheese), canned vegetables, biltong, shrimp/prawn
6	low in *moisture*, high in *available carbohydrate*, and thiamin	bread, breakfast cereals, cakes, cookies, puddings, pasta, pastries, maize and maize meal (stiff and crumbly), white rice, pies, dried fruit, jam/marmalade, honey, sugar, sweets
7	high in *cholesterol* and *vitamin D*	eggs and foods using eggs (e.g., custard, choux pastry), fortified milk powder, breastmilk substitutes, offal, tripe, battered/crumbed fish, fishcake made with egg, salmon, sardine, salad dressing
8	low in *zinc*, high in vitamin A, and *vitamin C*	fruit, vegetables, fruit juices, soft maize meal

Nutrients with absolute loadings >0.4 are indicated in italic.

**Table 4:** Food group percentage for each nutrient pattern (NP); *n* (%).

Food Group	Nutrient Pattern ^a^								Total
	NP 1	NP 2	NP 3	NP 4	NP 5	NP 6	NP 7	NP 8	
Cereals and Cereal Products	17 (17)		4 (3.64)	15 (17.05)	17 (21.52)	116 (70.73)	19 (21.35)	7 (2.16)	195 (20.08)
Vegetables	42 (42)	4 (23.53)	1 (0.91)	12 (13.64)	4 (5.06)	2 (1.22)		180 (55.56)	245 (25.23)
Fruit	3 (3)			1 (1.14)	1 (1.27)	20 (12.2)		107 (33.02)	132 (13.59)
Legumes and Legume Products	23 (23)			1 (1.14)		2 (1.22)			26 (2.68)
Nuts and Seeds	11 (11)			8 (9.09)		1 (0.61)			20 (2.06)
Milk and Milk Products					32 (40.51)	1 (0.61)	8 (8.99)		41 (4.22)
Eggs							27 (30.34)		27 (2.78)
Meat and Meat Products		9 (52.94)	89 (80.91)	15 (17.05)	2 (2.53)	2 (1.22)	3 (3.37)		120 (12.36)
Fish and Seafood		3 (17.65)	12 (10.91)	2 (2.27)	3 (3.8)		16 (17.98)		36 (3.71)
Fats and Oils				20 (22.73)	1 (1.27)	1 (0.61)	4 (4.49)		26 (2.68)
Sugar, Syrups and Sweets	1 (1)			3 (3.41)	1 (1.27)	11 (6.71)		1 (0.31)	17 (1.75)
Soups, Sauces, Seasonings and Flavouring	3 (3)	1 (5.88)		9 (10.23)	7 (8.86)	1 (0.61)	3 (3.37)	6 (1.85)	30 (3.09)
Beverages					8 (10.13)	3 (1.83)	1 (1.12)	15 (4.63)	27 (2.78)
Infant and Paediatric Feeds and Foods						3 (1.83)	7 (7.87)		10 (1.03)
Therapeutic/Special/Diet Products			4 (3.64)		2 (2.53)		1 (1.12)		7 (0.72)
Miscellaneous				2 (2.27)	1 (1.27)	1 (0.61)		8 (2.47)	12 (1.24)
Total	100 (100)	17 (100)	110 (100)	88 (100)	79 (100)	164 (100)	89 (100)	324 (100)	971 (100)

^a^ Blank cells represent 0 (0).

**Table 5:** Characterisation of food patterns (FP).

FP	Explained Variance (%)	Cumulative Explained variance (%)	Food Item with Absolute Loadings >0.05	Nutrients Which Scored High on Pattern
1	56.5	56.5	white and sweet potatoes, squash, celery, cucumber, cauliflower, brinjal, mushroom, green pepper, citrus fruits, stone fruits, and grapes in raw, canned, dried, and juice versions	potassium, magnesium, vitamin C
2	14.5	71.0	oats, rice, wheat, maize, barley, rye, wholewheat products, spinach and amaranth leaves, peas, green beans, berries (including pineapples), legumes and legume products, nuts and seeds, oyster, chocolate	manganese
3	10.4	81.4	milk and milk products (including foods made with milk and milk beverages), breastmilk substitutes, canned sardine, canned salmon	calcium
4	6.7	88.1	eggs and foods made with eggs (e.g., custard, choux pastry, sauces), meat and meat products, battered/crumbed fish	animal protein, cholesterol, phosphorous
5	3.9	92.0	bread, breakfast cereals, pastries, breadcrumbs, rusks made with white flour, canned vegetables, processed cheese, feta cheese, processed meat, meat pies, processed fish and seafood, sauces, icing for cakes	sodium
6	3.0	95.0	fortified maize meal, carrot (including carrot cake), pumpkin, butternut, hubbard squash, orange flesh sweet potato, leafy greens (e.g., lambquarters, sow thistle, cat’s whiskers leaves), apricot, mango, naartjie, beef kidney and liver, chicken liver and giblets, offal	vitamin A
7	2.4	97.4	soft maize meal, marrow, cabbage, brinjal, apple, pear, lemon, lime, fruit canned in syrup, trotters, tripe, miscellaneous (water, tea, coffee, alcohol)	moisture, plant protein, fatty acids, available carbohydrate, total fibre, iron, zinc, copper, thiamin, riboflavin, niacin, vitamin B_6_, vitamin B_12_, pantothenic acid, vitamin D, vitamin E

**Table 6:** Comparison between the South African food-based dietary guidelines and principal component analyses.

South African Food-Based Dietary Guidelines [4]	Corresponding Pattern
1. Enjoy a variety of foods.	The nutrient patterns obtained differ in nutritional composition.
2. Be active!	Not applicable
3. Make starchy foods part of most meals.	NP 6
4. Eat plenty of vegetables and fruit every day.	NP 8
5. Eat dry beans, split peas, lentils, and soya regularly.	NP 1
6. Have milk, maas, or yoghurt every day.	NP 5
7. Fish, chicken, lean meat, or eggs can be eaten daily.	NP 3, NP 7
8. Drink lots of clean, safe water.	Not applicable
9. Use fats sparingly. Choose vegetable oils, rather than hard fats.	NP 4
10. Use sugar and foods and drinks high in sugar sparingly.	NP 6
11. Use salt and food high in salt sparingly.	FP 5

**Table 7:** Comparison between the food categories targeted by the national sodium regulation and foods associated with FP 5.

Food Category as per the National Sodium Regulation [6]	Corresponding Foods Associated with FP 5
1. Bread	All bread types (pumpernickel, raisin, rye, sweetcorn, brown and white bread/rolls, breadcrumbs) except wholewheat bread/rolls
2. All breakfast cereals and porridges, whether ready-to-eat, instant or cook up, hot or cold	All breakfast cereals (puffed rice, puffed corn) except homemade muesli
3. All fat spreads and butter spreads	Mixed butter and hard margarine, brick/hard margarine, polyunsaturated margarine
4. Ready-to-eat savoury snacks, excluding salt-and-vinegar flavoured savoury snacks	Potato crisps
5. Flavoured potato crisps, excluding salt-and-vinegar flavoured potato crisps	
6. Flavoured, ready-to-eat, savoury snacks and potato crisps, salted and salt-and-vinegar only	
7. Processed meat, cured	Bacon, biltong, corned beef, ham, luncheon meat, meatloaf, pastrami, pork/beef sandwich spread
8. Processed meat, uncured	
9. Raw-processed meat sausages (all types) and similar products	Frankfurter, pepperoni, salami, sausages
10. Dry savoury soup powders	Soups, sauces
11. Dry gravy powders and savoury sauce powders	
12. Dry savoury powders with dry instant noodles	
13. Stock cubes, stock powders, stock granules, stock emulsions, stock pastes or stock jellies	

## Data Availability

No new data were created or analysed in this study. Data sharing is not applicable to this article.

## Supplementary Materials

The following are available online at https://www.mdpi.com/article/10.3390/nu13093194/s1, Figure S1: Screeplot for the principal component analysis of nutrients, Figure S2: Screeplot for the principal component analysis of food items.

## Appendix A

**Table A1:** Explained variance and rotated principal component loadings for the first eight nutrient patterns (NPs) identified by the analysis of nutrients.

Component	NP 1	NP 2	NP 3	NP 4	NP 5	NP 6	NP 7	NP 8
Explained variance (%)	14.9	10.1	9.6	9.3	9	8	7.6	4.9
Cumulative explained variance (%)	14.9	25	34.6	43.9	52.9	60.9	68.5	73.4
**Nutrients**								
Moisture	0.0084	0.0133	−0.0682	−0.2164	−0.0549	**−0.5215**	0.0035	−0.0133
Plant protein	**0.3867**	−0.0242	−0.0419	0.045	−0.0535	0.127	−0.0618	−0.0681
Animal protein	−0.1189	−0.0821	**0.4862**	0.0282	0.0676	−0.1463	0.1563	−0.1198
Saturated fatty acids	−0.1144	−0.0298	0.1156	**0.3581**	0.0453	0.0524	0.0531	−0.0496
Monounsaturated fatty acids	0.0125	−0.0222	0.0587	**0.5269**	−0.0025	−0.0095	−0.0378	−0.0669
Polyunsaturated fatty acids	0.0376	0.0355	−0.0781	**0.5328**	−0.0255	0.0038	−0.0191	0.0308
Cholesterol	−0.0064	0.0604	−0.025	−0.008	−0.028	−0.0622	**0.5883**	−0.0559
Carbohydrate, available	−0.0884	0.03	−0.1202	−0.142	−0.0158	**0.7057**	−0.0234	0.0669
Total dietary fibre	**0.3627**	−0.0134	−0.0418	−0.0491	−0.0484	0.0324	−0.0696	0.2578
Calcium	0.0703	0.0182	−0.1097	−0.0302	**0.5985**	−0.0281	0.0271	0.0584
Iron	0.2691	0.1124	0.0588	−0.0642	0.0972	0.1351	0.1487	−0.0592
Magnesium	**0.4515**	−0.0281	0.0234	0.0396	0.0523	−0.1023	0.028	0.001
Phosphorous	0.0421	0.0322	−0.0445	0.0024	**0.5864**	0.0047	0.01	−0.0436
Potassium	**0.3209**	−0.0459	0.1839	−0.0612	0.0478	−0.0488	0.0185	0.264
Sodium	−0.1109	−0.0525	0.1434	0.0302	**0.5085**	0.0352	−0.0727	−0.0058
Zinc	0.1675	0.1157	0.1339	−0.0286	−0.0123	−0.0914	−0.081	**−0.4198**
Copper	0.0509	**0.573**	−0.0206	0.0442	−0.0229	−0.032	−0.1268	−0.0586
Manganese	**0.4394**	−0.0077	−0.0655	0.0133	−0.0217	−0.0541	0.0014	−0.1554
Vitamin A (RE)	−0.018	**0.3839**	−0.0597	0.0217	0.0298	−0.0291	−0.0148	**0.3456**
Thiamin	0.1796	−0.0436	0.1771	−0.0286	−0.0454	**0.3188**	0.0556	−0.1524
Riboflavin	−0.0038	**0.3799**	0.0698	−0.0388	0.0141	0.1108	0.2049	−0.024
Niacin	−0.0464	0.0425	**0.5414**	0.0395	−0.0341	0.0695	−0.1054	−0.0615
Vitamin B_6_	0.1054	0.0272	**0.4134**	−0.0376	−0.0218	0.0863	−0.0923	0.1435
Vitamin B_12_	−0.0782	**0.5583**	0.0201	−0.0098	−0.0014	−0.0439	0.0106	−0.0643
Pantothenic acid	0.016	0.075	0.2889	−0.0291	−0.0384	−0.009	0.2697	0.154
Vitamin C	−0.0237	−0.0304	0.1344	0.0111	−0.0189	−0.073	−0.0439	**0.6353**
Vitamin D	−0.0103	−0.092	−0.0612	0.0223	0.0033	0.0671	**0.6351**	0.0271
Vitamin E	0.0854	0.0302	−0.1135	**0.4652**	−0.0215	−0.0181	0.1437	0.1294

Absolute loadings greater than 0.3 are shown in bold.

**Table A2:** Median (IQR) nutrient values by nutrient pattern (NP).

Nutrient	NP 1	NP 2	NP 3	NP 4	NP 5	NP 6	NP 7	NP 8	*p*-Value
Moisture (g)	69.9 (10.3–82.1)	71.8 (66.8–85.1)	61.4 (55.1–69.4)	50.1 (23.2–69.2)	76.4 (65.3–83.6)	24.9 (12–43.8)	72.2 (59.7–78)	85.7 (80.8–90.3)	<0.001
Plant protein (g)	5.3 (3.3–13.7)	0.7 (0.2–2)	0.7 (0.6–0.8)	1.9 (0.9–3.6)	0.5 (0.3–0.8)	3 (2.1–4.9)	1.1 (0.5–1.7)	0.9 (0.6–1.4)	<0.001
Animal protein (g)	1.4 (0–2.1)	20.4 (14.6–24.5)	24.7 (19.2–28.5)	2.5 (0.8–9.9)	3.8 (2.9–9.4)	1.4 (0.6–2.4)	9.1 (3.7–13.5)	0 (0–0.1)	<0.001
Saturated fatty acids (g)	0.2 (0.1–0.9)	1.1 (0.3–1.5)	3.1 (1.5–6.1)	6.1 (2.2–12)	1.6 (0.7–4.2)	1.6 (0.2–3.2)	2.7 (1.8–3.4)	0 (0–0.1)	<0.001
Mono-unsaturated fatty acids (g)	0.2 (0.1–1.8)	0.6 (0.2–1.4)	3.7 (2.1–6)	8.1 (3.4–16.6)	0.8 (0.3–3)	1.6 (0.3–4.7)	3.6 (1.6–4.4)	0 (0–0.1)	<0.001
Polyunsaturated fatty acids (g)	0.5 (0.2–2.8)	0.6 (0.3–1.1)	0.8 (0.3–2.4)	8.6 (3.5–11.7)	0.1 (0.1–0.8)	1.3 (0.4–3.2)	2.1 (0.7–3.6)	0.1 (0–0.2)	<0.001
Cholesterol (mg)	44.5 (8.1–49.4)	343 (165–426.8)	81 (57–97)	47.6 (21–91)	9.8 (6.5–37)	29.3 (18.5–49.5)	86.3 (62.6–280)	5.7 (2.2–7.6)	<0.001
Carbohydrate, available (g)	11.5 (4.8–23)	3.9 (2.5–5.9)	4.2 (1.6–6.3)	8.7 (3.6–19.8)	10.6 (4.9–15.4)	50.7 (38–67.5)	7 (2.4–14.9)	8.8 (4.3–14.7)	<0.001
Total dietary fibre (g)	5.5 (3.6–9.3)	1.1 (0.4–2.1)	0.7 (0.3–1.2)	1.4 (0.8–3.1)	0.2 (0.1–0.7)	1.7 (0.7–3.7)	0.3 (0.1–0.4)	1.8 (1.4–2.6)	<0.001
Calcium (mg)	54.5 (26.4–119.2)	12 (6–19)	15.2 (11–24)	21.4 (9.7–73.3)	118.9 (102.7–212)	27.7 (12.4–68.3)	65.8 (39–93.9)	15 (8–27)	<0.001
Iron (mg)	2.4 (1.5–4.7)	4.9 (1.1–6.2)	1.3 (0.8–2.2)	1 (0.5–1.7)	0.4 (0.2–0.8)	1.8 (0.9–2.9)	1.2 (0.7–1.8)	0.4 (0.3–0.6)	<0.001
Magnesium (mg)	59.5 (30–134)	16 (12–19.4)	24 (20–27)	11.5 (9–21.5)	14 (11.4–18.7)	18.2 (9.7–37.4)	11 (9.3–18.7)	11.4 (9–15)	<0.001
Phosphorous (mg)	92 (62–311.5)	244.7 (73–327)	196 (158.8–223)	79.1 (43.1–122.5)	110 (85.3–192.7)	75 (52.4–120)	142.8 (94.7–186)	26 (16–35)	<0.001
Potassium (mg)	362.5 (206–652)	229 (198–265)	287 (247–337)	143.4 (98–232.5)	141.2 (117–185)	120.9 (74.2–250.5)	128.4 (98–176)	186 (131–256.7)	<0.001
Sodium (mg)	17 (6–66.5)	85 (69–106.6)	81 (59–204.8)	59 (12.6–230.8)	113.2 (51.4–530)	68.5 (11–159.5)	119.4 (80.9–156.8)	7 (3–25.7)	<0.001
Zinc (mg)	1 (0.6–2.5)	3 (0.8–4.2)	2.5 (1.5–4.1)	0.4 (0.3–1.1)	0.5 (0.4–1)	0.5 (0.4–0.8)	0.6 (0.5–1.1)	0.2 (0.1–0.3)	<0.001
Copper (mg)	0.2 (0.2–0.5)	0.6 (0.4–4.5)	0.1 (0.1–0.2)	0.1 (0–0.1)	0 (0–0.1)	0.1 (0.1–0.2)	0.1 (0–0.1)	0.1 (0.1–0.1)	<0.001
Manganese (μg)	1189 (416.5–1894)	199 (133–345.5)	22 (14–70.1)	118.4 (29.5–254.4)	22.3 (4.9–66.5)	253.6 (156–360)	46.8 (38–110)	120.4 (70–200.6)	<0.001
Vitamin A (RE) (μg)	25 (4.5–163.5)	175.8 (41–1753)	14.6 (8–25.4)	64.1 (14.4–178.9)	43.1 (22.1–98.7)	55.9 (10.6–119.3)	68.6 (48–108.7)	22 (4.6–62.5)	<0.001
Thiamin (mg)	0.2 (0.1–0.4)	0.2 (0.1–0.2)	0.1 (0.1–0.2)	0.1 (0–0.2)	0 (0–0.1)	0.2 (0.1–0.3)	0.1 (0.1–0.1)	0 (0–0.1)	<0.001
Riboflavin (mg)	0.1 (0–0.2)	0.7 (0.2–2.8)	0.2 (0.1–0.3)	0.1 (0–0.2)	0.2 (0.1–0.2)	0.1 (0.1–0.2)	0.2 (0.2–0.3)	0 (0–0)	<0.001
Niacin (mg)	1 (0.6–2.4)	4.6 (1.2–9.6)	5.4 (4.2–7.9)	1.1 (0.4–2.8)	0.2 (0.1–0.5)	1.8 (0.7–2.6)	0.6 (0.1–1.5)	0.5 (0.3–0.7)	<0.001
Vitamin B_6_ (mg)	0.2 (0.1–0.4)	0.3 (0.1–0.7)	0.3 (0.2–0.4)	0.1 (0–0.1)	0 (0–0.1)	0.1 (0–0.2)	0 (0–0.1)	0.1 (0–0.1)	<0.001
Vitamin B_12_ (μg)	0.1 (0–0.3)	18.9 (8.8–48.8)	1.5 (0.5–2.2)	0.2 (0.1–0.4)	0.4 (0.3–0.6)	0.2 (0.1–0.3)	0.9 (0.4–1.5)	0 (0–0)	<0.001
Pantothenic acid (mg)	0.3 (0.1–0.8)	0.6 (0.2–3.3)	0.6 (0.3–1.1)	0.3 (0.1–0.5)	0.3 (0.3–0.4)	0.4 (0.2–0.4)	0.5 (0.4–1.1)	0.2 (0.1–0.3)	<0.001
Vitamin C (mg)	5.3 (1.2–14)	7.4 (2.7–10.4)	3.3 (1–8.3)	1.9 (0.9–4.3)	1 (0.8–2.2)	1 (0.2–3.8)	0.6 (0.4–4.4)	10.7 (4.4–24)	<0.001
Vitamin D (μg)	0.3 (0.3–0.9)	1.1 (0.6–1.2)	0.6 (0.2–1)	0.8 (0.4–1.9)	0.1 (0–0.5)	1 (0.5–1.7)	2.9 (1.4–5.9)	0.3 (0.2–0.3)	<0.001
Vitamin E (mg)	0.6 (0.2–2.2)	0.4 (0.1–0.8)	0.3 (0.2–0.5)	3.6 (1–6.9)	0.1 (0.1–0.6)	0.7 (0.3–1.3)	1.3 (0.7–3.4)	0.3 (0.1–0.6)	<0.001
