# Supplementary material for: Identifying Nutrient Patterns in South African Foods to Support National Nutrition Guidelines and Policies
Source: Nutrients. 2021 Sep 14;13(9):3194. doi: 10.3390/nu13093194 (PMC8465156; doi:10.3390/nu13093194)
Supplement: Supplementary file 1 [file nutrients-13-03194-s001.zip › nutrients-1367356-supplementary.pdf]

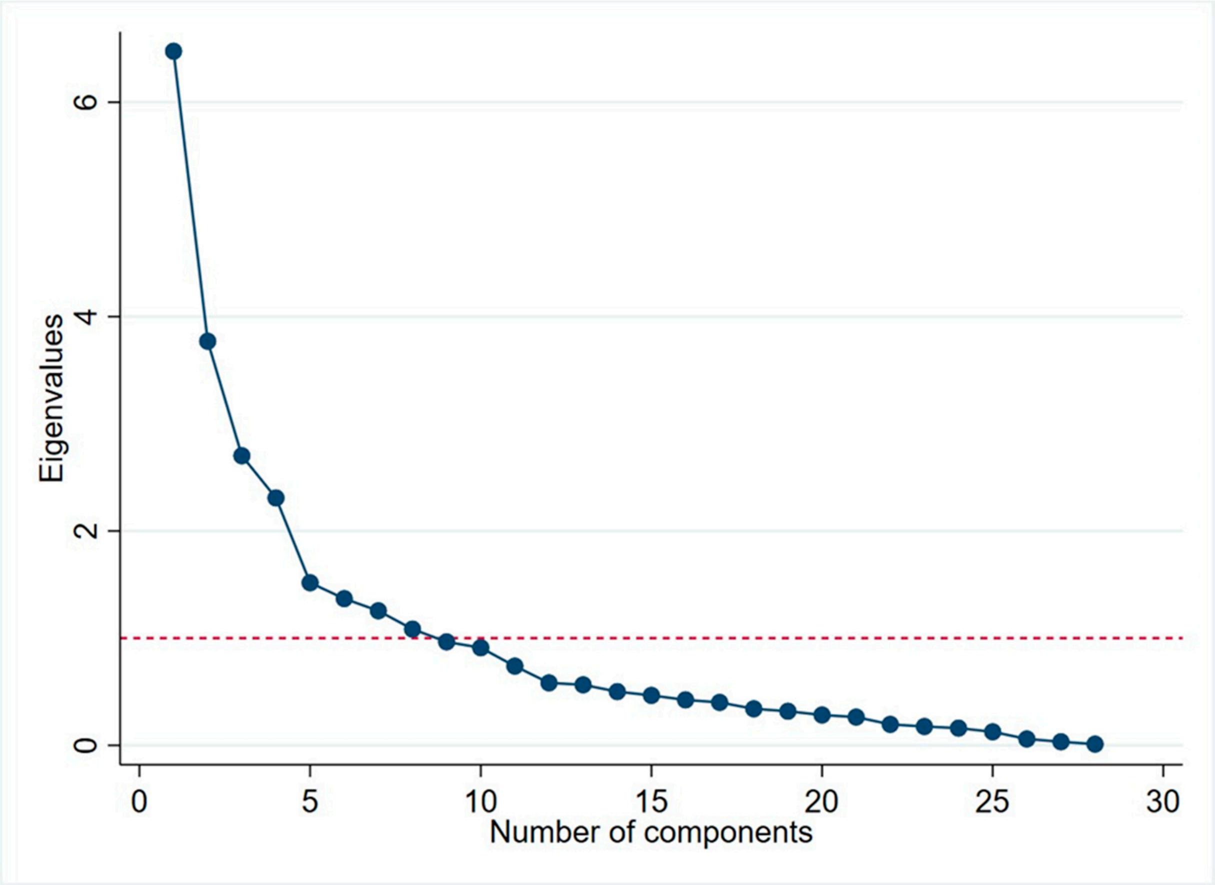

Figure S1. Screeplot for the principal component analysis of nutrients.

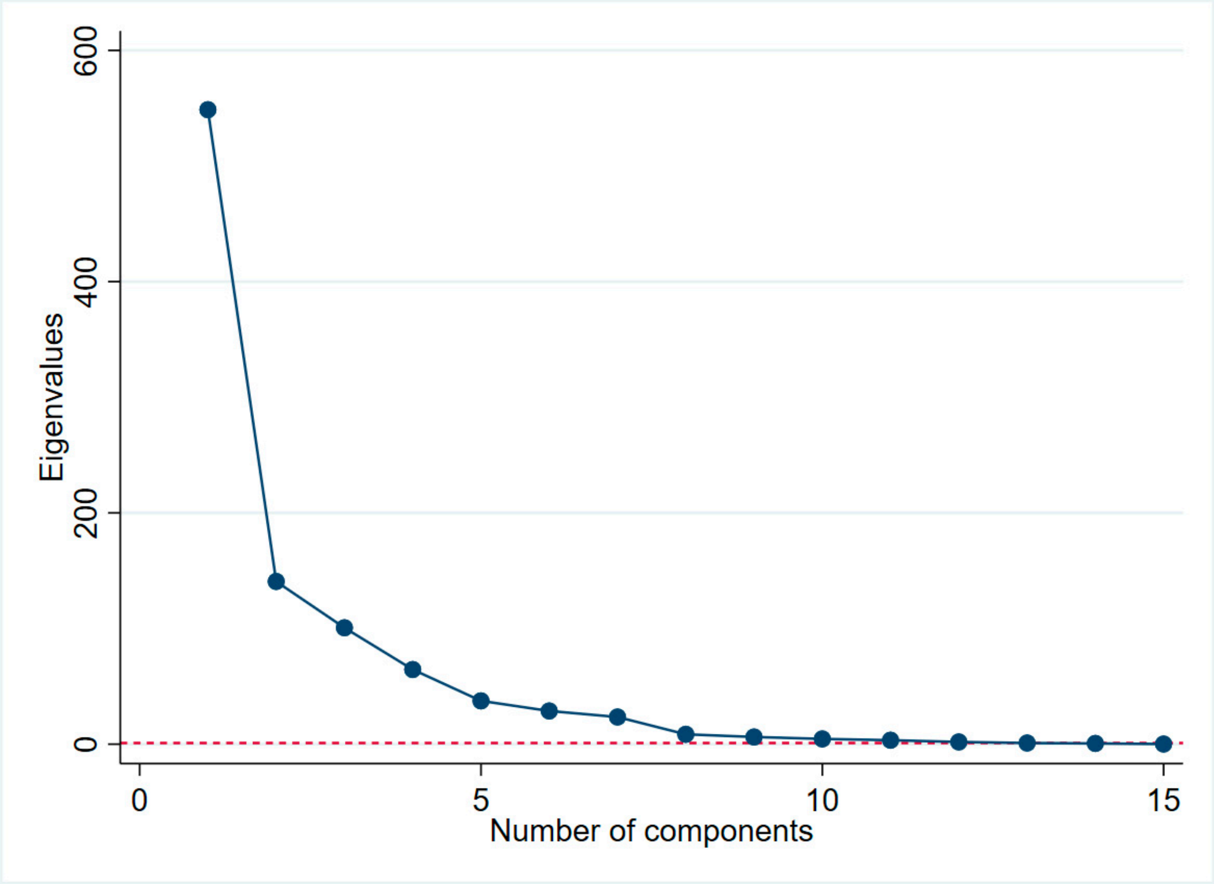

Figure S2. Screeplot for the principal component analysis of food items.
